# Hexokinase 2 Inhibition and Biological Effects of BNBZ and Its Derivatives: The Influence of the Number and Arrangement of Hydroxyl Groups

**DOI:** 10.3390/ijms23052616

**Published:** 2022-02-27

**Authors:** Karolina Juszczak, Anna Kubicka, Radosław Kitel, Grzegorz Dzido, Magdalena Łabieniec-Watała, Serafin Zawadzki, Agnieszka Marczak, Krzysztof Walczak, Karolina Matczak, Mateusz D. Tomczyk

**Affiliations:** 1 Department of Organic Chemistry, Bioorganic Chemistry and Biotechnology, Faculty of Chemistry, Silesian University of Technology, 44-100 Gliwice, Poland; karolina.juszczak@polsl.pl (K.J.); krzysztof.walczak@polsl.pl (K.W.); 2 Department of Medical Biophysics, Faculty of Biology and Environmental Protection, University of Lodz, 90-236 Lodz, Poland; anna.kubicka@edu.uni.lodz.pl (A.K.); magdalena.labieniec@biol.uni.lodz.pl (M.Ł.-W.); serafin.zawadzki@edu.uni.lodz.pl (S.Z.); agnieszka.marczak@biol.uni.lodz.pl (A.M.); 3 Department of Organic Chemistry, Faculty of Chemistry, Jagiellonian University, 30-060 Krakow, Poland; radoslaw.kitel@uj.edu.pl; 4 Department of Chemical Engineering and Process Design, Faculty of Chemistry, Silesian University of Technology, 44-100 Gliwice, Poland; grzegorz.dzido@polsl.pl

**Keywords:** hexokinase 2, Warburg effect, benitrobenzerazide, enzyme inhibition, aggregation

## Abstract

Hexokinase 2 (HK2), an enzyme of the sugar kinase family, plays a dual role in glucose metabolism and mediating cancer cell apoptosis, making it an attractive target for cancer therapy. While positive HK2 expression usually promotes cancer cells survival, silencing or inhibiting this enzyme has been found to improve the effectiveness of anti-cancer drugs and even result in cancer cell death. Previously, benitrobenrazide (BNBZ) was characterized as a potent HK2 inhibitor with good anti-cancer activity in mice, but the effect of its trihydroxy moiety (pyrogallol-like) on inhibitory activity and some cellular functions has not been fully understood. Therefore, the main goal of this study was to obtain the parent BNBZ (**2a**) and its three dihydroxy derivatives **2b**–**2d** and to conduct additional physicochemical and biological investigations. The research hypothesis assumed that the HK2 inhibitory activity of the tested compounds depends on the number and location of hydroxyl groups in their chemical structure. Among many studies, the binding affinity to HK2 was determined and two human liver cancer cell lines, HepG2 and HUH7, were used and exposed to chemicals at various times: 24 h, 48 h and 72 h. The study showed that the modifications to the structures of the new BNBZ derivatives led to significant changes in their activities. It was also found that these compounds tend to aggregate and exhibit toxic effects. They were found to contribute to: (a) DNA damage, (b) increased ROS production, and (c) disruption of cell cycle progression. It was observed that, HepG2, occurred much more sensitive to the tested chemicals than the HUH7 cells; However, regardless of the used cell line it seems that the increase in the expression of HK2 in cancer cells compared to normal cells which have HK2 at a very low level, is a serious obstacle in anti-cancer therapy and efforts to find the effective inhibitors of this enzyme should be intensified.

## 1. Introduction

In contrast to normally differentiated cells, which generate energy for cellular processes relying on oxidative phosphorylation (OXPHOS), most cancer cells instead use aerobic glycolysis, a phenomenon known as the Warburg effect. An enhanced glucose metabolic rate is required to meet the biosynthetic and bioenergetic demands of rapidly growing and proliferating cancer cells. In this context, aerobic glycolysis seems to be an inefficient way to generate energy as the maximum net yield is 2.73 moles of ATP *per mole of substrate* for pyruvate plus malate oxidation, while the maximum total yield is 33 moles of ATP for complete glucose oxidation [1]. However, cytosolic aerobic glycolysis can provide much more ATP *per unit of time* than mitochondrial oxidative phosphorylation since the speed of the cytosolic ATP generation is approximately 100 times faster than in mitochondria [2]. Cancer cells can compensate lower ATP production *per mole of substrate* using a pathway with high rate but low yield especially when they are in resource competition with normal cells that produce ATP at a higher yield but a lower rate. The outcome of this competition is determined by the highest growth rate and therefore by the highest rate of ATP production. Under these circumstances, HK2 plays a key role in tumor initiation and development as the first enzyme in the glycolysis pathway that phosphorylates glucose to G6P, introducing glucose into pathways required for anabolic activities in cancer cells [3]. Of the four HK isoforms in humans, HK2 is rarely expressed in normal tissues, except some insulin-sensitive tissues, but is highly expressed in several types of tumors, especially those that metastasize and have poor overall survival [4,5]. Furthermore, high level of HK2 is significantly associated with some phenotypes of tumor aggressiveness, such as large tumor size, positive lymph node metastasis and advanced clinical stage [6,7], while down-regulation of HK2 expression resulted in better efficacy of anti-cancer drugs [8,9] and promotes tumor radiosensitization [10,11]. In addition to satisfying energy requirements, HK2 expression protects cancer cells from apoptosis by binding to Voltage Dependent Anion Channel 1 on the outer mitochondrial membrane and by limiting reactive oxygen species (ROS) production in mitochondria by maintaining local ADP levels [12,13]. Therefore, HK2 represents a promising therapeutic target.

In recent years Liu et al. identified two classes of small-molecule HK2 inhibitors, i.e., benserazide and (E)-*N*′-(2,3,4-trihydroxybenzylidene) arylhydrazides by structure-based virtual screening of the ZINC database. Benserazide, a peripherally acting decarboxylase inhibitor used in combination with L-DOPA to the treatment of Parkinson’s disease, was identified as a promising HK2 inhibitor with an IC_50_ of 5.52 ± 0.17 μM and a dissociation constant (*K*_d_) of 149 ± 4.95 μM [14]. However, our attention was drawn to a second class of benserazide-related inhibitors developed by the same group that showed even better HK2 inhibition. Among them, the strongest activity was shown by benitrobenrazide (BNBZ) with an IC_50_ of 0.53 ± 0.13 µM and a reasonable selectivity of 3.2–10.9 for HK2 versus other isoenzymes, which placed it among the most potent non-glucose HK2 inhibitors [15]. Preliminary evaluation showed good anticancer activity in vitro and in vivo with negligible toxicity [16]. A common feature of this class of inhibitors is the presence of a 2,3,4-trihydroxybenzene moiety (pyrogallol-like), which is probably necessary to maintain inhibitory and cytotoxic activity in vitro. According to the docking model predicted by Liu et al. this fragment occupied the glucose binding site almost completely by overlapping the sugar ring, thus competing with glucose for the binding site. However, not all hydroxyl groups appear to be involved in the interaction with the binding site, and therefore, in searching for the possible mechanism(s) of action for this group of inhibitors, we proposed to check how changing the number and position of hydroxyl groups would affect the inhibitory activity and accompanying cellular effects.

**Scheme 1:**
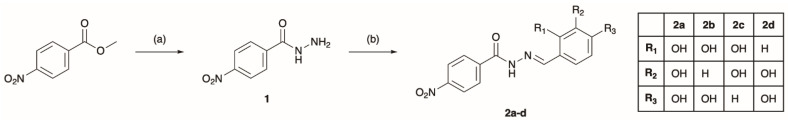
Synthesis of parent BNBZ (**2a**) and its dihydroxy derivatives **2b**–**2d**. *Reagents and conditions*: (**a**) 98% N_2_H_4_·H_2_O (3.0 eq.), MeOH, rt, 4 h; (**b**) ArCHO (1.2 eq.), MeOH, rt, 24 h.

Considering the above reports, we synthesized the parent inhibitor BNBZ, referred to as **2a**, and its three dihydroxybenzylidene derivatives **2b**–**2d** which differ in the arrangement of hydroxyl groups (Scheme 1). To test the hypothesis that the number and position of hydroxyl groups in BNBZ play an important role in the efficiency of HK2 inhibition, we conducted a series of in vitro studies using two human liver cancer cell lines, HepG2 and HUH7. Thus, the following detailed objectives were achieved: (1) the ability of the synthesized derivatives to inhibit HK2 activity, (2) evaluation of their cytotoxic and genotoxic properties, (3) evaluation of the generation of oxidative stress, (4) evaluation of nuclear DNA damage and cell cycle progression, and (5) evaluation of autophagy induction. Clarifying these points helped us to understand the general effect of BNBZ and its derivatives on HK2 activity and the response of the biological material used.

## 2. Results

### 2.1. Changes in the 2,3,4-Trihydroxybenzylidene Moiety Affect Binding to the Enzyme

**Figure 1:**
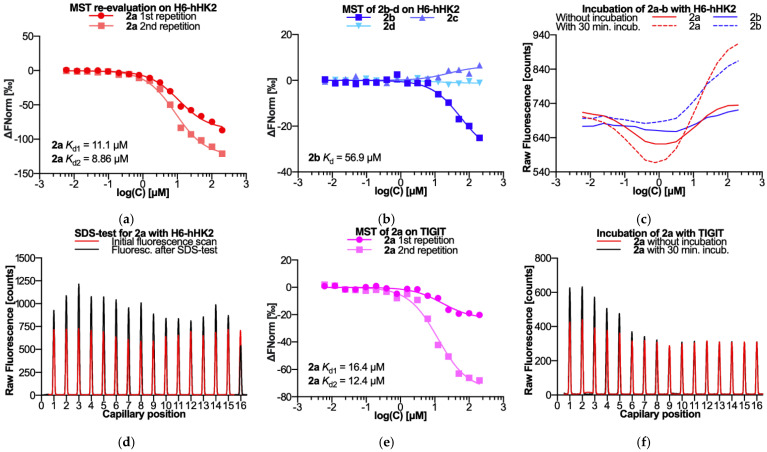
Evaluation of the binding affinity of (**a**) **2a** and (**b**) **2b**–**2d** to H6-hHK2. (**c**) Increasing trend profiles observed in initial capillary fluorescence before and after 30 min of H6-hHK2 incubation with **2a** and **2b**. (**d**) Initial capillary fluorescence before and after the **2a** SDS-test with H6-hHK2. (**e**) Evaluation of the binding affinity of **2a** to TIGIT. *K*_d_ values were automatically calculated by curve fitting and presented as mean. (**f**) Initial capillary fluorescence before and after 30 min incubation of TIGIT with **2a**. Capillaries have decreasing ligand concentration from left to right in serial 2-fold dilutions ranging from 200 to 0.006 µM.

First, we performed microscale thermophoresis (MST) measurements of the binding affinity of the synthesized compounds to H6-hHK2 (N-terminal His6-tagged human HK2), starting with a re-evaluation of the parent inhibitor **2a**, which is compound **3j** from the work of Liu et al. [17]. In two independent MST experiments, we found a *K*_d_ of 11.0 and 8.83 µM for **2a**, which are close to the *K*_d_ of 4.99 µM reported by the above authors (Figure 1a). The comparable *K*_d_ values indicate that despite the different method of labeling the protein (we used His-Tag labeling kit instead of amine-reactive kit as described in [17]), the dye adduct does not affect the interaction between the modified enzyme and the tested compound. All dihydroxy derivatives interact with H6-hHK2 much weaker than the trihydroxy **2a**, moreover, the overlap of the dose-response curves (Figure 1b) shows that not only the number but also the position of the hydroxyl groups can affect the binding affinity to the enzyme. Except for **2a**, only **2b** showed a moderate affinity for the enzyme with a *K*_d_ of 58.9 µM, while for **2c** and **2d** no binding affinity was detected, or the signal quality was not reliable. We also noted that the initial fluorescence levels were not constant in all samples. In particular, for **2a** and **2b**, we observed a clear upward trend in initial fluorescence starting at 12.5 µM and increasing with sample concentration and incubation time (Figure 1c). To verify whether the observed trend was due to ligand binding, we performed a denaturation assay (SDS-test) by heating the sample to 95 °C for 5 min in a solution containing 4% SDS and 40 mM DTT (Figure 1d). After denaturation, the differences in initial fluorescence still exceeded the recommended value of ± 10%, indicating that *K*_d_ values determined by this method may be overestimated due to sample adsorption or ligand-induced aggregation and should therefore be handled with caution. Additionally, we examined whether **2a** also binds to other, unrelated target proteins. T-cell immunoreceptor with Ig and ITIM domains (TIGIT) is a protein belonging to immune checkpoint receptors, and its overall fold is completely different from H6-hHK2, so it fits perfectly for this purpose. As we expected, **2a** behaved similarly when tested against TIGIT (Figure 1e). We observed a similar increasing trend in initial fluorescence when **2a** was incubated with TIGIT protein, supporting our concern that the results obtained at higher ligand concentrations might be falsified by aggregation events (Figure 1f).

### 2.2. Changes in the 2,3,4-Trihydroxybenzylidene Group Affect the Inhibitory Effect

To investigate the effect of the arrangement of the hydroxyl groups on the activity of the isolated and cellular enzyme, we compared the results of commercial in vitro and cell-based HK2 activity assays. In these enzymatic assays, glucose is phosphorylated to G6P by HK2, which is then oxidized by glucose-6-phosphate dehydrogenase to form NADH. The resulting NADH activates the probe by reducing its colorless form to a colored product with strong absorbance at 450 nm, which is proportional to HK2 activity present. As positive controls, we used two most commonly reported HK2 inhibitors, 3-bromopyruvate (**3BP**) and 2-deoxyglucose (**2DG**), which differ significantly in their mechanism of enzyme inhibition. **2DG** is a glucose analog that is phosphorylated by HK2 to form 2DG-6-phosphate, which cannot be further metabolized, so it accumulates inside cells and inhibits HK2, presumably through product-mediated inhibition [17,18]. Unlike **2DG**, **3BP** is a potent alkylating reagent that inactivates HK2 and possibly dissociates this enzyme from the mitochondria [19,20,21]. To determine the inhibitory effect of **2a**–**2d** in cells, we chose two liver cancer cell lines, HepG2 and HUH7. Cell-based assays were performed at IC_50_ concentrations that resulted in 50% inhibition of proliferation of the respective cell lines (these concentrations are determined and presented later in this article).

Since we noticed sings of aggregation in previous experiments, we tested different sample formulations to better investigate this phenomenon and its effect on enzyme inhibition in vitro. Previously, Shoichet’s group found that aggregate formation can be disturbed by adding low concentrations of detergents to biochemical media, thus preventing aggregate-enzyme interactions but not well-behaved inhibitor-enzyme interactions [22]. They also found that a small addition of DMSO increases the apparent solubility of compounds, thus increasing their critical aggregation concentration, but does not contribute to aggregate-mediated enzyme inhibition [23]. Following these procedures, we added 0.1% Tween 20 to the in vitro buffer and prepared two series of samples by: (1) dissolving solid samples directly in the buffer, or (2) diluting concentrated DMSO stocks into the buffer, so that the final DMSO concentration does not exceed 1%.

**Figure 2:**
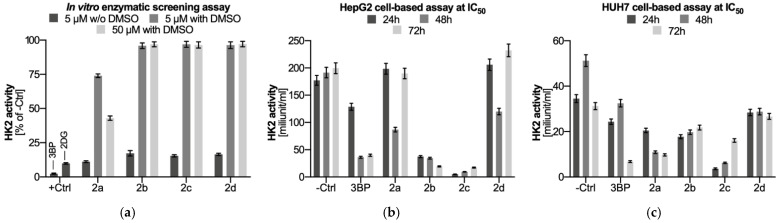
(**a**) In vitro enzyme inhibition assay results. **3BP** (2.5 ± 0.4%) and **2DG** (9.9 ± 0.5%) are positive controls (+Ctrl), both compounds were used at 5 µM. The negative control is the corresponding assay system without inhibitor, whose activity is assumed to be 100%. **3BP** and **2DG** samples were prepared in assay buffer containing 0.1% Tween 20. Changes in HK2 activity in (**b**) HepG2 and (**c**) HUH7 cell lines after 24 h, 48 h and 72 h incubation with the test compounds at IC_50_ concentrations. **3BP** at 100 µM was used as a positive control. Data are presented as milliunits of enzyme activity per milliliter of reaction mixture. Data are presented as mean and SD, *n* = 3. The significance of differences was estimated based on multifactor ANOVA and post-hoc Tukey tests. Statistical analysis for cellular assay: HepG2+2a: *p* < 0.05: control vs. IC_50_ 48 h; HepG2+2b: *p* < 0.001: control vs. IC_50_ 24 h, IC_50_ 48 h and IC_50_ 72 h; HepG2+2c: *p* < 0.001: control vs. IC_50_ 24 h, IC_50_ 48 h and IC_50_ 72 h; HepG2+2d: *p* < 0.01: control vs. IC_50_ 48 h; HUH7+2a: *p* < 0.05: control vs. IC_50_ 24 h; *p* < 0.001 control vs. IC_50_ 48 h and IC_50_ 72 h; HUH7+2b: *p* < 0.01: control vs. IC_50_ 48 h; *p* < 0.001 control vs. IC_50_ 24 h and IC_50_ 72 h; HUH7+2c: *p* < 0.05: control vs. IC_50_ 72 h; *p* < 0.001 control vs. IC_50_ 24 h and IC_50_ 48 h; HUH7+2d: *p* < 0.001 control vs. IC_50_ 48 h. For more experimental details please see “Materials and Methods”.

All 5 µM samples prepared in DMSO-free buffer inhibited HK2 with similar efficiency to 11.2–17.3% of the negative control activity (Figure 2a). In contrast, in the DMSO-containing series, only **2a** retained inhibitory activity, while no significant inhibition was observed for the reaming derivatives at any of the concentrations. These results support a nonspecific mechanism of action for these compounds and suggest that the inhibition may be due to aggregate formation (the presence of which is confirmed later in this article). Small colloidally aggregating molecules (SCAMs) acting through this mechanism appear to be relatively common in virtual and experimental screening libraries and even among true leads. In fact, some 2-hydroxyphenylhydrazones closely related to BNBZ have already been described by Shoichet’s group as aggregate-forming promiscuous enzyme inhibitors [22,23,24,25]. In cellular assays, the inhibitory activity decreases in the order **2c** >> **2b** > **2a** > **2d** for the HepG2 line (Figure 2b), and **2c** >> **2a** > **2b** > **2d** for the HUH7 line (Figure 2c). The highest inhibitory effect was observed for **2c**, which almost completely inhibited cellular HK2 after 24 h of incubation in both cell lines; however, **2c** was tested at relatively high concentrations of 213.7 and 302.1 µM, depending on the cell line. In the case of the HepG2 line, significant inhibition is also shown by **2b**, which at 2.8 µM reduced HK2 activity to a similar level as **3BP** at 100 µM.

### 2.3. The Tested Compounds Tend to Aggregate

**Figure 3:**
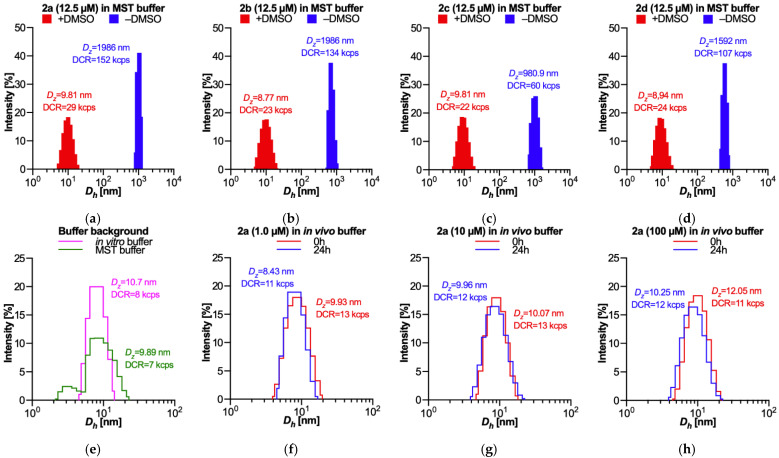
Summary of size distribution of hydrodynamic diameters (*D*_h_), Z-average hydrodynamic diameters (*D*_z_) and derived count rates (DCR) for 12.5 µM solutions of (**a**) **2a**, (**b**) **2b**, (**c**) **2c** and (**d**) **2d** in MST buffer (25 mM phosphate buffer, pH 8.0, 500 mM NaCl and 0.05% Tween-20). (**e**) DLS signals from buffers and other background noises. DLS signals before and after 24 h incubation of (**f**) 1.0 µM, (**g**) 10 µM and (**h**) 100 µM solutions of **2a** in in vitro assay buffer containing 0.1% Tween-20. No other peaks above 100 nm were detected. All samples obtained from DMSO stocks were measured at a final concentration of 1% DMSO; no serial dilutions were performed.

To determine if **2a**–**2d** also belongs to SCAMs, we investigate their aggregation behavior using dynamic light scattering (DLS), a well-established biophysical technique used to determine particle size in solution [26]. We first examined the aggregation of 12.5 µM solutions of **2a**–**2d** prepared in MST buffer, since during the MST measurements most of the tested samples showed irregular fluorescence behavior starting from this concentration, which became more pronounced as the concentration increased. Despite the relatively high concentration of detergent in the MST buffer (0.05% Tween 20), all DMSO-free (–DMSO) samples formed clearly detectable particles with a Z-average diameter (*D_z_*) of 980.9–1986 nm and a derived count rate (DCR) of 60–152 kcps, by DLS (Figure 3). The DCR value represents the theoretical count rate at 100% laser power, so for the same sample, higher DCR values should indicates a higher concentration, larger particles, or a combination of both. The DLS experiment was then repeated with samples prepared by diluting the DMSO stock (+DMSO) to see if aggregation could be disrupted by transferring a well-dissolved concentrated DMSO solution to a buffer containing detergent. Indeed, we observed that the large colloids were disrupted and disappeared completely, and instead we obtained *D*_z_ values ranging from 8.77 to 9.39 nm, which may indicate the presence of smaller, monodisperse colloids (Figure 3a–c). It should be noted, however, that according to the manufacturer’s recommendations, scattering from water for particles smaller than 10 nm should give a count rate in excess of 10 kcps to obtain reliable results. Measurement of the MST buffer background gave *D*_z_ = 9.89 nm and DCR = 7 kcps (Figure 3d), which are close to the values determined for dilute DMSO stocks. Such results suggest two possibilities: (1) The significantly lower DCR values for samples with DMSO (22–29 kcps), which are still higher than those obtained for the buffer alone (7 kcps), indicate the presence of some aggregation, but negligible compared to samples without DMSO (60–152 kcps); (2) *D*_z_ values for samples containing DMSO (8.77–9.39 nm) are similar to those obtained for the buffer background (9.89 nm), which may indicate the complete disappearance of aggregation. Due to such problems in interpreting the results for dilute solutions, the method we used can only be useful for general comparison of the degree of aggregation between samples, but it does not allow the determination of the critical aggregation concentration or other advanced parameters.

Similar results were obtained for **2a** at different concentrations prepared by diluting the DMSO stock solution in in vitro assay buffer containing 0.1% Tween 20 (Figure 3f–h). The reduction in size and number of aggregates may explain the attenuation or loss of inhibitory activity observed during the in vitro experiments (Figure 2a). It was previously speculated that transfer of small organic compounds from polar organic solvents such as DMSO to the aqueous environment could promote aggregation, however, Shoichet’s group demonstrated that DMSO is not necessary to promote this phenomenon and that aggregation-based inhibition can also occur under aqueous conditions without DMSO [24]. Here, we have shown that preparing solutions from DMSO stocks can even stop this process and help eliminate false-positive results. Interestingly, despite the lack of serial dilution, *D_z_* (9.93–12.05 nm) and DCR (11–13 kcps) values are similar for all concentrations tested, which differ by several orders. Under these conditions, DCR values were even slightly lower than in MST buffer, which may be due to the effect of higher detergent concentration or lower ionic strength on aggregation. We further examined whether small colloids of **2a**, which could potentially be present in the screening buffer, are stable over time or whether they clump together and grow. After 24 h of incubation at room temperature, no significant changes in *D*_z_ and DCR values were observed, further indicating that there is very little or no aggregation. The simple DLS-based aggregation assay presented here can be useful as a preliminary test in biological assays to select appropriate conditions for compound preparation, e.g., to determine whether DMSO should necessarily be used to prepare solutions for testing.

### 2.4. The Tested Compounds Are Cytotoxic to HepG2 and HUH7cells

**Figure 4:**
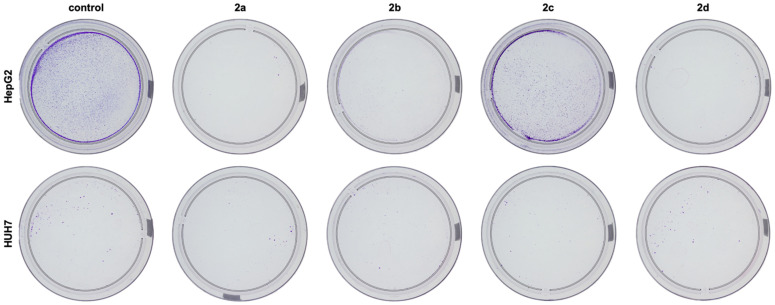
Colony forming ability (clonogenicity) in HepG2 and HUH7 cell lines treated with **2a**–**2d** at their IC_50_ for 72 h. Photographs were taken after staining the cells with crystal violet. After cell fixation and staining with crystal violet, photographs were taken and the obtained data were quantified using ImageJ software (see Table 1).

**Figure 5:**
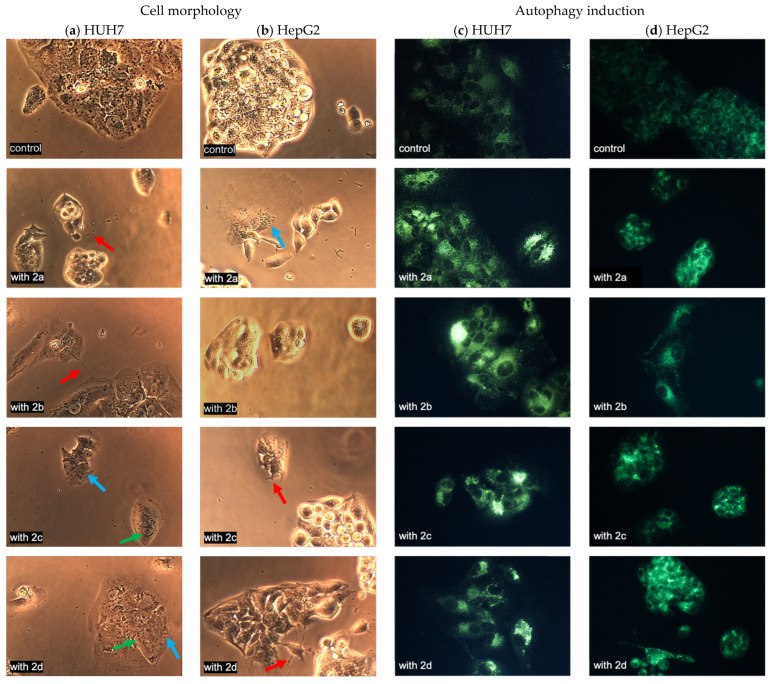
Left panel: Morphology of (**a**) HUH7 and (**b**) HepG2 cells after incubation for 72 h with tested compounds **2a**–**2d** applied at their IC_50_ (shown in Table 1). Changes in morphology are indicated by arrows in the figures: the red arrow indicates the cytoplasmic linkages, the green arrow indicates abnormal cell nuclei, and the blue arrow indicates swellings of the cell’s cytoplasm. Right panel: Induction of autophagy in (**c**) HepG2 and (**d**) HUH7 cells after incubation for 72 h with tested compounds **2a**–**2d** applied at their IC_50_ (shown in Table 1). The first row of images shows control cells, while the following figures show autophagosomes formed after treatment with the tested compunds. Images were taken using an Olympus IX70 microscope at 150× magnification.

In order to determine the antiproliferative properties of the tested compounds, the MTT assay was used. IC_50_ values for HepG2 and HUH7 cell lines treated with **2a**–**2d** for 72 h are shown in Table 1. In brief, HUH7 cells showed less sensitivity to most of the tested compounds (except **2c**) than HepG2 cells. The strongest cytotoxic effect was found for **2a** and **2b** against HepG2, while **2c** showed significantly lower activity against both cell lines, which was reflected not only in cytotoxicity but also in the clonal assay, a method of determining cell survival based on the ability of a single cells to form colonies. The results in Figure 4 show that the colony forming ability of HUH7 and HepG2 cells was reduced after treatment with the tested compounds; **2a** reduced the growth of HUH7 cells to about 22.5%, while the growth of HepG2 cells was inhibited to 15.8%; **2b** inhibited the growth of HUH7 and HepG2 cells to 25.6% and 9.9%, respectively. In contrast, **2d** reduced the growth of HUH7 and HepG2 cells to approx. 40% and 13%, respectively. The morphology of cells treated with compounds **2a**–**2d** at their IC_50_ for 72 h is also shown in Figure 5. The tested compounds induced changes in both HUH7 and HepG2 cells when compared to untreated cells. The effect of the tested agents is evident by a significant increase in cell volume and the appearance of numerous cytoplasmic junctions signaling the induction of apoptosis. However, the presence of abnormal cellular nuclei within the cell interior and the presence of numerous swellings in the cytoplasm may indicate the induction of other types of cell death, such as autophagy or mitotic catastrophe [27,28].

### 2.5. The Tested Compounds Disturb the Oxidoreductive Balance of Cells

**Figure 6:**
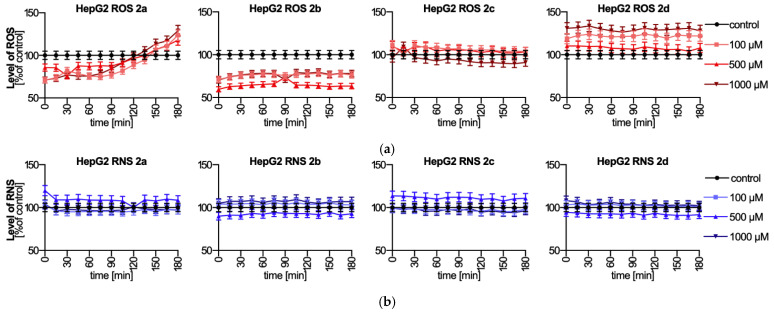
Comparison of changes in (**a**) ROS and (**b**) RNS levels in HepG2 cells treated with three different concentrations (100, 500 and 1000 µM) of **2a**–**2d** for 0–180 min compared to ROS and RNS levels in a control sample (100%). ROS and RNS formation were tested overtime, in the range of 0–180 min after the adding of the tested compounds. Results are presented as mean ± SD from three independent experiments. The significance of differences was estimated based on multifactor ANOVA and post-hoc Tukey tests. Statistical analysis for ROS level: HepG2+2a: *p* < 0.01: control vs. 120 min (100, 500 and 1000 µM); *p* < 0.001 control vs. 0–105 min and 165–180 min (100, 500 and 1000 µM); HepG2+2b: *p* < 0.001: control vs. 0–180 min (100, 500 and 1000 µM); HepG2+2d: *p* < 0.001: control vs. 0–180 min (100 and 1000 µM). Statistical analysis for RNS level: HepG2+2a: *p* < 0.05: control vs. 0 min (500 µM); HepG2+2c: *p* < 0.05: control vs. 0–180 min (500 µM). For more experimental details please see “Materials and Methods”.

**Figure 7:**
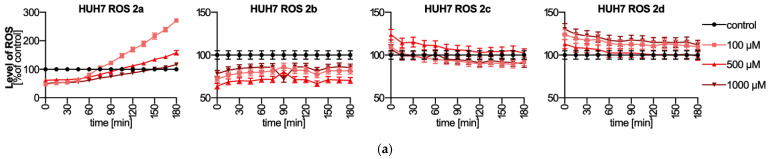

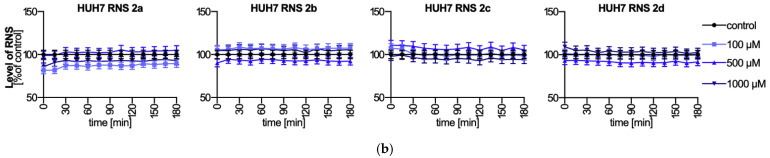
Comparison of changes in (**a**) ROS and (**b**) RNS levels in HUH7 cells treated with three different concentrations (100, 500 and 1000 µM) of **2a**–**2d** for 0–180 min compared to ROS and RNS levels in a control sample (100%). ROS and RNS formation were tested overtime, in the range of 0–180 min after the adding of the tested compounds. Results are presented as mean ± SD from three independent experiments. The significance of differences was estimated based on multifactor ANOVA and post-hoc Tukey tests. Statistical analysis for ROS level: HepG2+2a: *p* < 0.05: control vs. 60 min (100, 500 and 1000 µM); control vs. 90 min (100 µM); *p* < 0.01: control vs. 135–150 min (500 µM); *p* < 0.001 control vs. 0–45 min (100, 500 and 1000 µM); control vs. 105–180 min (100 µM); control vs. 165–180 min (500 µM); HepG2+2b: *p* < 0.001: control vs. 0–180 min (100, 500 and 1000 µM); HepG2+2c: *p* < 0.05: control vs. 0–30 min (500 µM); HepG2+2d: *p* < 0.01: control vs. 0–120 min (100 and 1000 µM). Statistical analysis for RNS level: HepG2+2a: *p* < 0.01: control vs. 0–45 min (100 µM). For more experimental details please see “Materials and Methods”.

Changes in the levels of reactive oxygen species (ROS) and reactive nitrogen species (RNS) in cells exposed to the tested compounds were assessed spectrofluorimetrically. The respective compounds had the same effect on the redox balance in both cell lines, with the ROS level strongly dependent on the number and position of hydroxyl groups (Figure 6a and Figure 7a), while the RNS level did not change significantly regardless of the tested compound (Figure 6b and Figure 7b). Previously, Rushmore et al. observed a structure-activity relationship for simple polyphenols tested on the HepG2 line. They found that oxidative stress was induced by 1,2-diphenol (catechol) and 1,2,3-trihydroxybenzene (pyrogallol), but not by 1,3-diphenol (resorcinol) [29]. Indeed, we observed that administration of the parent compound **2a** with a pyrogallol-like hydroxyl configuration and its catechol-like derivative **2d** increased ROS levels in both cell lines, while the prooxidant activity of **2c** was much lower and resorcinol-like derivative **2b** even led to decreased ROS levels. The reason for the lower prooxidant activity of **2c** compared to **2d**, with the same hydroxyl configuration, is probably the lack of extended conjugation through the C=N double bond, analogous to the effect responsible for the differences in the self-oxidation rate of some flavones [30]. These findings are consistent with literature data on pyrogallol and catechol-type compounds, which are known to be hepatoxic compounds with strong ability to induce oxidative stress [31,32], DNA fragmentation and apoptosis in various cells [33,34].

### 2.6. The Tested Compounds Inhibit Autophagy

**Figure 8:**
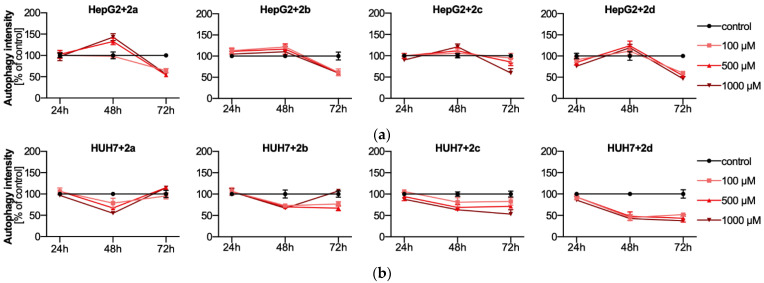
Autophagy induction in (**a**) HepG2 and (**b**) HUH7 cells treated with **2a**–**2d** at three concentrations (100, 500 and 1000 µM) for 24 h, 48 h and 72 h. Results are presented as mean ± SD from three independent experiments, control was taken as 100%. The significance of differences was estimated based on multifactor ANOVA and post-hoc Tukey tests. HepG2+2a: *p* < 0.05: control vs. 100 µM (48 h and 72 h); 100 µM vs. 500 µM (48 h); *p* < 0.01: control vs. 500 µM (48 h); control vs. 1000 µM (48 h and 72 h); 100 µM vs. 500 µM (72 h); 100 µM vs. 1000 µM (48 h); 500 µM vs. 1000 µM (48 h); *p* < 0.001: control vs. 500 µM (72 h); HepG2+2b: *p* < 0.05: 24 h vs. 48 h (1000 µM); *p* < 0.01: 48 h vs. 72 h (100 µM); HepG2+2c: *p* < 0.05: 48 h vs. 72 h (1000 µM); HepG2+2d: *p* < 0.05: control vs. 500 µM (48 h); 24 h vs. 72 h (1000 µM); *p* < 0.01: 24 h vs. 72 h (100 µM); *p* < 0.001: control vs. 100 µM (72 h); control vs. 500 µM (72 h); control vs. 1000 µM (72 h); 48 h vs. 72 h (100 µM); 24 h vs. 48 h (500 µM); 500 µM vs. 1000 µM (72 h); 24 h vs. 48 h (1000 µM); HUH7+2a: *p* < 0.05: control vs. 100 µM (48 h); 48 h vs. 72 h (100 µM); 24 h vs. 72 h (1000 µM); *p* < 0.01: 100 µM vs. 1000 µM (48 h); *p* < 0.001: control vs. 500 µM (48 h); control vs. 1000 µM (48 h); 24 h vs. 48 h (100 µM); 24 h vs. 48 h (500 µM); 48 h vs. 72 h (500 µM); 500 µM vs. 1000 µM (72 h); 24 h vs. 48 h (1000 µM); HUH7+2b: *p* < 0.05: control vs. 100 µM (48 h); 48 h vs. 72 h (100 µM); *p* < 0.01: 100 µM vs. 1000 µM (48 h); *p* < 0.001: control vs. 500 µM (48 h); control vs. 1000 µM (48 h); 24 h vs. 48 h (100 µM); 24 h vs. 48 h (500 µM); 48 h vs. 72 h (500 µM); 24 h vs. 48 h (1000 µM); HUH7+2c: *p* < 0.05: control vs. 100 µM (48 h); 500 µM vs. 1000 µM (72 h); *p* < 0.01: control vs. 100 µM (72 h); *p* < 0.001: control vs. 500 µM (48 h); control vs. 1000 µM (48 h); control vs. 500 µM (72 h); control vs. 1000 µM (72 h); 100 µM vs. 1000 µM (72 h); 24 h vs. 48 h (500 µM); 48 h vs. 72 h (500 µM); 24 h vs. 48 h (1000 µM); 24 h vs. 72 h (1000 µM); HUH7+2d: *p* < 0.05: control vs. 100 µM (48 h); 500 µM vs. 1000 µM (72 h); *p* < 0.01: control vs. 100 µM (72 h); *p* < 0.001: control vs. 500 µM (48 h); control vs. 1000 µM (48 h); control vs. 500 µM (72 h); control vs. 1000 µM (72 h); 24 h vs. 48 h (500 µM); 24 h vs. 72 h (500 µM); 24 h vs. 48 h (1000 µM); 24 h vs. 72 h (1000 µM). For more experimental details please see “Materials and Methods”.

The monodansylcadaverine staining technique was used to determine the intensity of the autophagy process. This compound is a specific marker for autophagosomes. Lack of both oxygen and nutrients can lead to cell death through apoptosis or necrosis. According to the literature, one of the functions of autophagy is to protect the cell under stressful conditions by “self-digesting” elements inside the cell [35]. Our results indicate that the tested compounds did not inhibit the autophagy process in HepG2 and HUH7 cells (Figure 8). Only in the case of the HepG2 line, an increase in autophagy is observed after 48 h, but a decrease in autophagy activity after 72 h, was observed. This indicates that the DNA damage generated by the compounds is so intense that the autophagy process cannot protect the cell. Recently, there has been an intensified discussion on the phenomenon of “autophagy switch” [36].

Autophagy is a catabolic process involving the supply of nutrients in response to energy deficiency, a process that promotes cell survival under stress conditions by degrading damaged organelles and acquiring metabolites. The phenomenon of autophagy is regulated by the availability of nutrient and thus plays a key role in the metabolism of glucose, which is the primary energy source used by the vast majority of cells. The involvement of autophagy in cell death is highly desirable for effective anti-cancer therapy. Autophagy, which does not show any cytoprotective effect, allows for the intensification of the cytotoxic effect of the applied substances, contributing, inter alia, to for the induction of apoptosis. When disturbed cellular metabolism (e.g., by inhibition of the normal function of HK2) leads to intensive removal of damaged cell structures by autophagy, a parallel induction of apoptosis is often observed [37]. The observed gradual inhibition of autophagolysosome formation in cells treated with the tested compounds may indicate that also in this case we are dealing with the phenomenon of lack of cytoprotective effect by autophagy and induction of the apoptosis process.

### 2.7. The Tested Compounds Damage DNA and Disrupt the Progression of Cell Cycle

**Figure 9:**
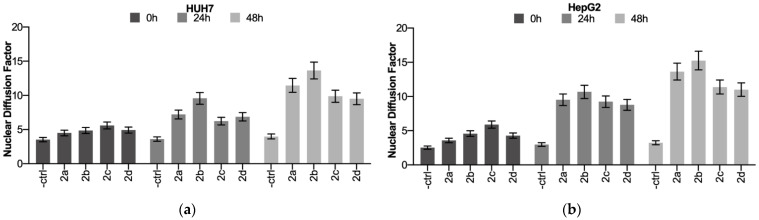

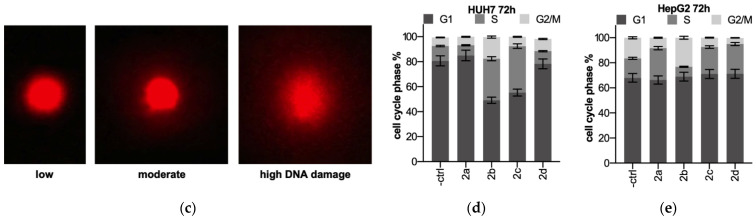
Measurement of DNA damage generated by the tested compounds in (**a**) HUH7 and (**b**) HepG2 cell lines. DNA damage was presented as the Nuclear Diffusion Factor. (**c**) Photographs of a representative cell nucleus observed during the DNA damage test. Small, medium, and large DNA damage can be seen, visible in the photograph as a gradually growing halo around the undamaged DNA. Images were taken using an Olympus IX70 microscope at 400× magnification. Cell-cycle distribution of (**d**) HUH7 and (**e**) HepG2 cell lines. The cell cycle was analyzed cytometrically after 72 h of treatment with **2a**–**2d** used at their IC_50_. The percentage of DNA at a particular cell-cycle phase was calculated from DNA histograms. Single experiments were carried out at least in triplicate, each with 3 to 4 repeats. Data were expressed as mean and SD. The significance of differences was estimated based on multifactor ANOVA and post-hoc Tukey tests. Statistical analysis for Fast HALO Assay: HepG2+2a: *p* < 0.001: control vs. IC_50_ (24 h and 48 h); IC_50_ 0 h vs. IC_50_ 24 h; IC_50_ 0 h vs. IC_50_ 48 h; IC_50_ 24 h vs. IC_50_ 48 h; HepG2+2b: *p* < 0.001: control vs. IC_50_ (0 h, 24 h and 48 h); IC_50_ 0 h vs. IC_50_ 24 h; IC_50_ 0 h vs. IC_50_ 48 h; IC_50_ 24 h vs. IC_50_ 48 h; HepG2+2c: *p* < 0.001: control vs. IC_50_ (0 h, 24 h and 48 h); IC_50_ 0 h vs. IC_50_ 24 h; IC_50_ 0 h vs. IC_50_ 48 h; IC_50_ 24 h vs. IC_50_ 48 h; HepG2+2d: *p* < 0.001: control vs. IC_50_ (0 h, 24 h and 48 h); IC_50_ 0 h vs. IC_50_ 24 h; IC_50_ 0 h vs. IC_50_ 48 h; IC_50_ 24 h vs. IC_50_ 48 h; HUH7+2a: *p* < 0.001: control vs. IC_50_ (24 h and 48 h); IC_50_ 0 h vs. IC_50_ 24 h; IC_50_ 0 h vs. IC_50_ 48 h; IC_50_ 24 h vs. IC_50_ 48 h; HUH7+2b: *p* < 0.01: control vs. IC_50_ 0 h; *p* < 0.001: control vs. IC_50_ (24 h and 48 h); IC_50_ 0 h vs. IC_50_ 24 h; IC_50_ 0 h vs. IC_50_ 48 h; IC_50_ 24 h vs. IC_50_ 48 h; HUH7+2c: *p* < 0.001: control vs. IC_50_ (0 h, 24 h and 48 h); IC_50_ 0 h vs. IC_50_ 48 h; IC_50_ 24 h vs. IC_50_ 48 h; HUH7+2d: *p* < 0.05: control vs. IC_50_ 0 h; *p* < 0.001: control vs. IC_50_ (24 h and 48 h); IC_50_ 0 h vs. IC_50_ 24 h; IC_50_ 0 h vs. IC_50_ 48 h; IC_50_ 24 h vs. IC_50_ 48 h. Statistical analysis for cell cycle distribution: HepG2+2a: *p* < 0.001: control vs. S phase and G2/M phase; HepG2+2b: *p* < 0.001: control vs. S phase and G2/M phase; HepG2+2c: *p* < 0.001: control vs. S phase and G2/M phase; HepG2+2d: *p* < 0.001: control vs. S phase and G2/M phase; HUH7+2a: *p* < 0.05: control vs. G1 phase; *p* < 0.001: control vs. S phase; HUH7+2b: *p* < 0.001: control vs. G1 phase, S phase and G2/M phase; HUH7+2c: *p* < 0.001: control vs. G1 phase and S phase; HUH7+2d: *p* < 0.05: control vs. S phase; *p* < 0.001: control vs. G2/M phase. For more experimental details please see “Materials and Methods”.

One of the major markers of cell damage by a potentially cytotoxic compound is DNA damage. In fact, DNA damage can have many causes, but most often it results from an increase in oxidative stress in cells [38]. Human liver cancer cells were incubated with the tested compounds at IC_50_ concentration for 24 h, 48 h and 72 h, and the extent of DNA damage was assessed using the Fast Halo Assay (FHA) method. We showed that all tested compounds caused significant DNA damage which increased with incubation time (Figure 9a–c). Since the tested compounds did not generate high oxidative stress, the observed DNA damage is probably a consequence of either a secondary phenomenon or the result of a direct interaction of the tested compounds or their metabolites with DNA [39].

Cell cycle cytometric analysis showed that all tested compounds caused arrest of HepG2 and HUH7 cells in G1 phase, however this effect was mainly observed in HUH7 cells (Figure 9d,e). The arrest in G1 phase is clearly observed for **2b**–**2d** after 72 h at IC_50_ concentration. In contrast, moderate G1 arrest occurred with all tested compounds in HepG2 cells. Overall, the results achieved are consistent with those obtained for DNA damage. The effect obtained against cancer cells may be related to the fact that inhibition of HK2 may lead to the formation of mitochondrial permeability pores and unblock the recruitment of mitochondrial proapoptotic proteins such as Bax and Bak [40].

## 3. Discussion

We started by synthesizing the parent HK2 inhibitor BNBZ, referred to as **2a**, and its three dihydroxy derivatives, **2b**–**2d** (Scheme 1). Using MST, we examined the binding affinity of the synthetized compounds to HK2 and found that only **2a** exhibited a reasonable *K*_d_ of 8.83–11.0 µM (Figure 1a), indicating its moderate interaction with the enzyme. However, this interaction was not highly selective, as **2a** binds with similar potency to TIGIT (*K*_d_ = 12.4–16.4 µM), a protein unrelated to HK2 (Figure 1e). During MST, we also noticed irregular fluorescence behavior at high concentrations of the tested samples, which combined with their poor target selectivity, prompted us to investigate their aggregation behavior in more detail (Figure 1c–f). Using the DLS method, we found that indeed **2a**–**2d** have a strong tendency to form micrometric colloids (*D*_z_ = 980.9–1986 nm) in aqueous media in the micromolar range (Figure 3), which may explain the initial false-positive results obtained in the in vitro enzyme inhibition assay for DMSO-free samples (Figure 2a). Based on the procedures developed by Shoichet’s group, we were able to significantly reduce the level of aggregation (*D*_z_~10 nm) among all compounds tested (Figure 3), which in turn resulted in a complete disappearance of in vitro inhibitory activity of dihydroxy derivatives **2b**–**2d**. Only trihydroxy **2a** retained its inhibitory activity in vitro. These results are consistent with previously determined binding affinities and together may indicate that a pyrogallol-like moiety is necessary to maintain inhibitory activity in this group of compounds. We suppose that despite the suppression of aggregation, its effect on enzyme behavior remains significant, especially at higher ligand concentrations in MST studies, as seen in Figure 1c.

The aggregation-based mechanism may explain not only the unusual behavior observed during our enzymatic and MST studies, but also the relatively flat structure-activity relationship seen among the pyrogallol-based library of 24 compounds obtained by Liu et al. [15]. On the other hand, the same research group was able to determine the enzymatic kinetic of HK2 at low concentrations of **2a** (0.125–0.5 µM) and show that this molecule is ATP-incompetent but glucose-competitive inhibitor [16]. If the results of this experiment were affected by aggregates, their effect would extend to all kinetic experiments because the entire enzyme molecule would be involved in nonspecific interactions with aggregates, but no such effect was observed. Since these studies were performed with relatively low ligand concentrations delivered from DMSO stocks, the critical aggregation concentration may not have been exceeded and aggregates may not have formed even in the absence of detergents. It is worth noting that of the more than 50 compounds studied by the Shoichet’s group, the critical aggregate concentration for the strongest aggregates started at about 0.5–1.2 µM, and below these values most of the studied compounds appear to remain in monomeric form. In our studies on compound **2a**, we found the disappearance of large aggregates even at a concentration of 100 µM (Figure 3h) in samples containing DMSO and a small addition of detergent; however, it should be noted that the method we used has some limitations, which does not allow us to unequivocally state the complete absence of aggregates.

The results concerning HK2 inhibition in the cellular environment also provide interesting insights. As we expected in the initiation stage of the study, the effects of potential inhibitors vary depending on whether they interact with the isolated enzyme or with the enzyme contained in the intact cell. When studying HK2, it is very possible to encounter the problem that HK2 is a part of larger complexes composed of various interacting components (including proteins) deposited on the outer mitochondrial membrane. Thus, new places of interaction may be available, not necessarily located on HK2 itself, that can become targets for small-molecule chemicals and, upon binding with them, lead to “inhibition” of the enzyme in a non-classical manner—e.g., by detaching from the mitochondrial membrane and depriving it of convenient access to ATP. For these reasons, the effects of potential HK2 inhibitors should always be tested both in vitro and using cell-based assays. Several compounds are now known that inhibit the effects of HK2 in cells but have no effect on the isolated enzyme; these include methyl jasmonate, which has been shown to detach HK2 from the mitochondrial membrane [41,42,43]. Our research group has also found several such “inhibitors” and we are currently studying them to understand their mechanism of action and preferential strategies for HK2 inhibition in the cellular environment. Relating these considerations to the results presented herein, it can be noted that the reference HK2 inhibitor, **3BP**, and the parent compound **2a** inhibit the enzyme both in vitro and inside cells, whereas its dihydroxy derivatives **2b**–**2d** fails in vitro but some of them show inhibitory activity inside cells. A completely different issue is **2c**, which although inhibits HK2 to about 4–11% of control, but at the same time achieves much less cytotoxicity compared to **2a**, **2b** and **2d** (see Table 1). This is interesting because it may indicate that inhibition of HK2 in cancer cells does not necessarily lead to its death.

The biological activities of the tested compounds were analyzed in many areas, including inhibition of HK2 activity in cells, cytotoxicity, oxidative stress and induction of cell death caused by damage to cellular components. Since we suspected the tested compounds of having prooxidant effects, which is also suggested in the literature [29], we decided to check what other effects accompany their administration and whether they are responsible for the observed cytotoxicity of the compounds. Zheng et al. postulate that the cellular redox imbalance is a result of HK2 disconnection from the mitochondrial membrane, which leads to increased leakage of ROS from mitochondria [16]. It should be noted, however, that mitochondria are not the only source of ROS in the cell. ROS can also be generated, for example, in the reduction of molecular oxygen by myeloperoxidase and NADPH oxidase—key enzymes involved in the first line of defense against pathogens. Even the molecules of the tested compounds, after getting inside the cell, can undergo many transformations, the side effect of which may be oxidative stress.

When analyzing the biological activity of chemical substances in terms of their potential use in anticancer therapy, particular attention should be paid to the cytotoxic properties of the tested substances and the induction of cell death. Our team focused in particular on the process of autophagy, which can both lead to cell death and be the cell’s defensive mechanism against the cytotoxic effects of exogenous substances [37]. Autophagy is a catabolic process involving the uptake and degradation of the cell contents. This process is exacerbated by nutrient deficiencies and promotes survival by eliminating damaged organelles and recovering metabolites. In principle, glucose deficiency should inhibit the activity of the mTORC1 kinase complex in cells, thereby reducing anabolic metabolism and stimulating autophagy [44]. Roberts et al. made the unexpected observation that **2DG** could prevent the induction of autophagy in response to glucose deficiency in cardiac myocytes. Therefore, it was expected that **2DG** treatment would lower ATP levels, leading to AMPK activation and the subsequent induction of autophagy via ULK1 phosphorylation [45].

In our study, there was little or no induction of autophagy after 48 h (Figure 8a). This may indicate that the DNA damage, which increases with exposure to the tested compounds (Figure 9a–c), is so high that the autophagy process is unable to remove it and thus protect the cell from death. Other studies show that autophagy may play a dual role in cancer, which may, as already mentioned, help cancer cells survive under stressful conditions such as hypoxia or nutrient deprivation [46]. Correlations between apoptosis and autophagy have been observed, although whether autophagy induces or inhibits apoptosis depends on the cell type, character and duration of the stimulus. Due to the different interactions of autophagy and apoptosis in cancer, they can be divided into a synergistic effect, a promoting effect and an antagonistic effect [47]. If apoptosis is promoted, autophagy may enhance it. The cell cycle arrest in HepG2 and HUH7 cells in the G1 phase may indicate activation of the apoptotic death pathway (Figure 9d,e).

## 4. Conclusions

The parent compound **2a** was selected by Liu et al. as potent HK2 inhibitor by virtual screening (VS) and subsequent in vitro structure-activity optimization [15]. vs. has become a key component of successful drug discovery at both an industrial and academic level, but as Baell and Holloway mentioned, it should come as no surprise that most of hits from vs. campaigns comprise predominantly false positives hits [48]. As they write, compounds can give false positives results if, for example, they interfere with binding interactions by forming aggregates, react with proteins, or directly interfere with assay signaling. The resulting consequences can be avoided already at the vs. stage by using appropriate substructure filters that remove problematic compounds with unwanted functionalities. To identify problematic features of the tested compounds, we performed ADME studies using the SwissADME predictor, a free web tool to assess the pharmacokinetics and drug-likeness of small molecules [49]. As a result, we identified at least three unwanted functionalities: (1) the hydroxyphenylhydrazone moiety, which is known chelator [50] and macromolecule aggregator in bioassays [24], (2) catechol and pyrogallol moieties capable of forming transient but highly reactive quinone methides [51], and (3) the potentially mutagenic and highly cytotoxic nitrobenzyl group [52]. Although many studies have shown that hydroxyphenylhydrazones belong to PAINS and SCAMs and thus should be removed from vs. results, examples of successful inhibitor design with these compounds can also be found in the literature. Some of these examples come from Jiang’s group, which has developed several *β*-hydroxyacyl-acyl carrier protein dehydratase (FabZ) inhibitors based on the 3,5-dibromo-2,4-dihydroxybenzylidenehydrazide moiety [53,54]. The crystal structure determination showed that the relatively rigid benzylidenehydrazide linker is an essential component of the inhibitor structure, which facilitate its binding by reducing entropy loss while fitting into the sterically restrained FabZ binding pocket. Hence, removal of hydrazones in early lead structure discovery may be unnecessary and likely limits chemical diversity. For the reason given above, we cannot simply conclude that the parent compound **2a** is unsuitable for further research and structure modification during medicinal chemistry optimization. In our opinion, further development of BNBZ as a lead structure for new HK2 inhibitors, if undertaken, should focus on the elimination of problematic functionalities and careful assessment of physicochemical and biological properties, especially aggregation, genotoxicity, and overall reactivity of this group of compounds. Instead, we can conclude that according to the research hypothesis, the number and arrangement of hydroxyl groups affects the inhibitory and biological properties, and the pyrogallol group is crucial for maintaining the inhibitory activity of BNBZ.

The results of our work and the problems we encountered during its implementation fit well with the ever-growing trend of paying attention to whether the obtained results are not false positives. We are becoming more familiar and sensitive to the problems associated with the design of biologically active compounds, especially at the academic level, and are getting better at dealing with them. Here we would particularly like to recommend the work of Shoichet’s group, as it is the leading research group on SCAMs. The studies presented here focusing on the assessment of the biological activity of potential HK2 inhibitors are currently preliminary studies. To fully answer questions regarding practical application of the tested compounds, further attempts should be made to determine their exact mechanism of action and all the side effect associated with their in vivo administration. Special attention should also be paid to their formulation, as BNBZ and its derivatives show a strong tendency to aggregate, which may have a great impact on the results of biological studies.

## 5. Materials and Methods

### 5.1. General Information

All reagents and solvents were obtained from commercial sources and used without further purification. Reactions were monitored by TLC analysis using silica gel 60 F_254_ plates (Merck, Darmstadt, Germany). Column chromatography was performed on silica gel 60 (Merck). ^1^H- and ^13^C-NMR spectra were recorded on a model 600 NMR spectrometer (Varian, Palo Alto, CA, USA) at 600 and 150 MHz, respectively. Peak multiplicity is expressed as follows: s = singlet, d = doublet, t = triplet, dd = doublet of doublets. NMR chemical shifts are given in ppm (δ), relative to residual non-deuterated solvents as internal standard, coupling constants (*J*) are given in Hz. Melting points (mp) were determined using a Boethius PHMK apparatus (VEB Analytik, Jena, Germany) and were not corrected. High resolution electrospray ionization mass spectroscopy (ESI-HRMS) analyzes were performed using a Waters Xevo G2 Q-TOF apparatus (Waters, Milford, MA, USA). 

### 5.2. Chemical Synthesis

Scheme 1 shows the general method for the synthesis of 4-nitrobenzohydrazide (**1**), the parent compound BNBZ (**2a**), and its derivatives **2b**–**2c**. Hydrazine monohydrate (98%, 4.50 mL, 90.0 mmol) was added in one portion to a solution of methyl 4-nitrobenzoate (5.42 g, 30.0 mmol) in methanol (40.0 mL) and the solution was stirred at room temperature for 4 h. The reaction mixture was poured into water (400 mL) and the resulting light-yellow precipitate of **1** was filtered, air dried, and used without further purification. The appropriate aldehyde (3.30 mmol) was added to a suspension of **1** (500 mg, 2.80 mmol) in methanol (15.0 mL) and stirred at room temperature for 24 h. The resulting precipitates of **2a**–**2d** were filtered and refluxed twice in ethanol (15.0 mL) to remove unreacted starting materials.

### 5.3. Spectroscopic Data

*4-Nitrobenzohydrazide* (**1**): Yield 65%. Mp. 210 °C. ^1^H-NMR (DMSO-d_6_): δ 4.64 (s, 2H), 8.06 (d, *J* = 9.0 Hz, 2H), 8.30 (d, *J* = 9.0 Hz, 2H), 10.13 (s, 1H). ^13^C-NMR (DMSO-d_6_): δ 123.45, 128.35, 138.93, 148.82, 163.81. HRMS (ESI-TOF): *m*/*z* calcd for C_7_H_8_O_3_N_3_ [M + H]^+^ 182.0566; found: 182.0073.

*(E)-N′-(2,3,4-trihydroxybenzylidene)-4-nitrobenzohydrazide* (**2a**): Yield 53%. Mp. 248 °C. ^1^H- NMR (DMSO-d_6_): δ 6.42 (d, *J* = 8.4 Hz, 1H), 6.85 (d, *J* = 8.4 Hz, 1H), 8.18 (d, *J* = 8.4 Hz, 2H), 8.39 (d, *J* = 8.4 Hz, 2H), 8.51 (s, 1H), 8.54 (br. s, 1H), 9.55 (br. s, 1H), 11.43 (br. s, 1H), 12.24 (br. s, 1H). ^13^C-NMR (DMSO-d_6_): δ 107.68, 110.67, 121.13, 123.67, 129.01, 132.64, 138.53, 147.53, 148.52, 149.20, 150.92, 160.72. HRMS (ESI-TOF): *m*/*z* calcd for C_14_H_10_N_3_O_6_ [M − H]^−^ 316.0575; found: 316.1483.

*(E)-N′-(2,4-dihydroxybenzylidene)-4-nitrobenzohydrazide* (**2b**): Yield: 42%. Mp. >250 °C. ^1^H- NMR (DMSO-d_6_): δ 6.34 (d, *J* = 2.4 Hz, 1H), 6.38 (dd, *J* = 8.4, 2.4 Hz, 1H), 7.35 (d, *J* = 8.4 Hz, 1H), 8.17 (d, *J* = 8.4 Hz, 2H), 8.38 (d, *J* = 8.4 Hz, 2H), 8.55 (s, 1H), 10.01 (br. s, 1H), 11.30 (br. s, 1H), 12.18 (br. s, 1H). ^13^C-NMR (DMSO-d_6_): δ 102.55, 107.75, 110.36, 123.57, 128.98, 131.13, 138.62, 149.17, 149.79, 159.45, 160.70, 160.92. HRMS (ESI-TOF): *m*/*z* calcd for C_14_H_10_N_3_O_5_ [M − H]^−^ 300.0626; found: 300.1619.

*(E)-N′-(2,3-dihydroxybenzylidene)-4-nitrobenzohydrazide* (**2c**): Yield 76%. Mp. >250 °C. ^1^H- NMR (DMSO-d_6_): δ 6.76 (t, *J* = 7.8 Hz, 1H), 6.89 (dd, *J* = 8.4, 2.4 Hz, 1H), 7.03 (dd, *J* = 8.4, 2.4 Hz, 1H), 8.19 (d, *J* = 8.4 Hz, 2H), 8.39 (d, *J* = 8.4 Hz, 2H), 8.66 (s, 1H), 9.30 (br. s, 1H), 10.92 (br. s). 12.38 (br. s, 1H) ^13^C-NMR (DMSO-d_6_): δ 117.50, 118.70, 119.13, 119.77, 123.60, 129.10, 138.40, 145.56, 146.10, 149.28, 149.64, 161.06. HRMS (ESI-TOF): *m*/*z* calcd for C_14_H_10_N_3_O_5_ [M − H]^−^ 300.0626; found: 300.1625.

*(E)-N′-(3,4-dihydroxybenzylidene)-4-nitrobenzohydrazide* (**2d**): Yield 50%. Mp. >250 °C. ^1^H- NMR (DMSO-d_6_): δ 6.81 (d, *J* = 7.8 Hz, 1H), 6.97 (dd, *J* = 8.4, 1.8 Hz, 1H), 7.28 (d, *J* = 1.8 Hz, 1H), 8.15 (d, *J* = 8.4 Hz, 2H), 8.30 (s, 1H), 8.37 (d, *J* = 8.4 Hz, 2H), 9.38 (br. s, 2H), 11.90 (br. s, 1H). ^13^C-NMR (DMSO-d_6_): δ 112.68, 115.50, 120.81, 123.51, 125.39, 128.97, 139.27, 145.67, 148.19, 149.05, 149.36, 161.02. HRMS (ESI-TOF): *m*/*z* calcd for C_14_H_10_N_3_O_5_ [M − H]^−^ 300.0626; found: 300.0625.

### 5.4. Protein Expression and Purification of H6-hHK2

The human hexokinase 2 construct (hHK2, aa 17-917; based on Uniprot entry P52789) was subcloned into pET-15b vector. The construct was transformed in *E. coli* BL21(DE3) and cultured at 37 °C in 1.0 L TB media supplemented with ampicillin (100 μg/mL) until an OD_600_ of 1.0 was reached. Protein expression (bearing N-terminal His6-tag) was induced by adding 1.0 mM isopropyl-β-d-thiogalactopyranoside (IPTG) and shaking the cultures overnight at 16 °C. The following day, the cells were harvested (20 min, 4000 rpm) and resuspended in ice-cold lysis buffer (10 mM Tris-HCl pH 8.0, 200 mM NaCl, 1.0 mM DTT, 1.0 mM PMSF). The lysate was clarified by centrifugation (30 min, 15,000 rpm) and soluble fraction of proteins was loaded onto DEAE weak anion exchanger column (GE Healthcare, Chicago, IL, USA) connected to ÄKTA Pure System (GE Healthcare). The protein was purified using a gradient of 200–800 mM NaCl over 10 cv. Fractions containing hHK2 were pooled and dialyzed against 2.0 l of binding buffer (50 mM Tris-HCl pH 8.0, 500 mM NaCl, 25 mM imidazole) overnight. The following day, the protein was loaded onto HiPrep IMAC pre-equilibrated with binding buffer (50 mM Tris-HCl pH 8.0, 500 mM NaCl, 25 mM imidazole). After removal of unbounded proteins, hHK2 was eluted from the column using elution buffer (50 mM Tris-HCl pH 8.0, 500 mM NaCl, 200 mM imidazole). Fractions containing hHK2 were pooled, concentrated, and loaded onto a Superdex S200 10/30 gel-filtration column equilibrated with gel filtration buffer (25 mM phosphate, pH 8.0, 500 mM NaCl). Peak fractions containing hHK2 were pooled and concentrated in Amicon Ultra-15 concentrator units (30,000 Da cut-off; Millipore, Burlington, MA, USA) to final concentration of 1.0 mg/mL (10 μM).

### 5.5. Microscale Thermophoresis

The experiments were performed on Monolith NT.115 (NanoTemper Technologies, Munich, Germany). Fluorescence labelling of H6-HK2 with the RED-Tris-NTA dye was performed according to the manufacturer’s protocol of Monolith His-Tag Labeling Kit RED-Tris-NTA 2nd Generation (NanoTemper Technologies). Prior to labelling, protein was diluted to 200 nM in 25 mM phosphate, 500 mM NaCl and 0.05% Tween-20. An equal volume of 100 nM dye was prepared, mixed with the H6-HK2 protein solution, and incubated with for 30 min at room temperature. Next, the labelled protein was centrifuged for 10 min at 16,900 rpm. After labelling, the protein was stored on ice. Compounds stocks (2× concentrated, 400 µM) were prepared in 25 mM phosphate buffer, 500 mM NaCl, 1% DMSO and 0.05% Tween-20 (*v*/*v*), pH 8.0. Sixteen 10 µL of two-fold serial dilutions of each compound were prepared in two 8-well PCR strips. Next, 10 µL of 80 nM protein was added to each well. This led to a final HK2 concentration of 40 nM. The highest concentration of compound in the assay was 200 µM. Compound-protein solutions were centrifuged for 10 min and then loaded into MST Premium capillaries. Measurements were made at 80% IR laser power and medium LED intensity. Data were analyzed using NanoTemper Analysis software, from which F_norm_ values (F_norm_ = F_hot_/F_cold_) as well as initial fluorescence were exported to excel files and evaluated in GraphPad Prism software (GraphPad Software, San Diego, CA, USA). Dissociation constants (*K*_d_) values were also calculated using the GraphPad Prism software.

### 5.6. Cell Culture

All studies were performed on two well-differentiated liver cancer cell lines, HepG2 (ATCC, Manassas, VA, USA) and HUH7 (ECACC, Salisbury, UK). Both cell lines were cultured as monolayers in Dulbecco’s modified Eagle’s medium (DMEM; Lonza, Bornem, Belgium), supplemented with 10% (*v*/*v*) fetal bovine serum (Gibco, Waltham, MA, USA) and 1% (*v*/*v*) penicillin/streptomycin mixture (10,000 U/mL penicillin and 10 mg/mL streptomycin; Lonza) in a 100% humidified atmosphere of 5% CO_2_ and 95% air at 37 °C. The cell cultures were periodically screened for Mycoplasma contamination. The cell cultures were kept in the exponential growth phase by regular passaging of the cells three times a week with a 0.25% trypsin/EDTA mixture (Lonza, Bornem, Belgium).

### 5.7. Cell Morphology

Changes in morphology of cells treated with the tested compounds were assessed as described elsewhere [55]. The day before the experiments, cells were seeded at the appropriate density onto 35 mm diameter dishes. The next day, the tested compounds were added at IC_50_ concentrations and cells were incubated for 72 h. At the end of incubation, the medium was aspirated, the cell monolayers were washed twice with pre-warmed PBS, and finally HBSS solution was added. Qualitative analysis of morphological changes was performed using an IX70 fluorescence microscope (Olympus, Tokyo, Japan) at 150× magnification.

### 5.8. Cytotoxicity Assay

The cytotoxic activity of tested compounds was examined by the standard MTT spectrophotometric method described elsewhere [56]. Briefly, exponentially growing cells were seeded into each well of a 96-well microplate (5 × 10^3^/well). After 24 h, tested compounds were added to the appropriate wells in a series of concentrations, and the cells were incubated for 72 h. After treatment with the tested compounds, the medium was removed and a solution of MTT (3-[4,5-dimethylthiazol-2-yl]-2,3-diphenyltetrazolium bromide) was added at a final concentration of 0.5 mg/mL. After 4 h of incubation, the MTT solution was replaced with DMSO to dissolve the formed violet formazan crystals in metabolically viable cells. The plates were gently shaken at room temperature to allow complete dissolution of the formazan and read at 540 nm with a microplate reader. The experiment was repeated at least three times under the same conditions. The cytotoxicity of the tested compounds was assessed on the basis of their IC_50_ values, i.e., the concentration causing a 50% reduction in cell viability as compared to untreated (control) cells which were arbitrary taken as 100%. IC_50_ values were calculated using the GraphPad Prism software.

### 5.9. Hexokinase Activity Assays

Hexokinase activity in cells was measured using the Hexokinase Colorimetric Assay Kit (Cat. No. MAK091-1KT; Merck). Briefly, at appropriate time points, control and treated cells were released from monolayers by trypsinization, resuspended in PBS, and washed twice. Subsequent steps of the enzymatic activity analysis were carried out according to the manufacturer’s instructions. In vitro hexokinase activity was measured using the Human Hexokinase 2 (HK2) Inhibitor Screening Kit (Cat. No. K713; BioVision, Milpitas, CA, USA) according to the manufacturer’s instructions. No direct activation of the probe was observed, nor its indirect activation caused by the reduction of NAD to NADH by tested compounds. Aqueous solutions for in vitro studies were prepared by stirring the tested compounds for 16 h in screening kit buffer containing 0.05% Tween 20 followed by incubation for 8 h at 37 °C. The resulting solutions were then filtered through 0.22 µm syringe filters. Concentrated stocks of **2a**–**2d** were prepared at 0.5 and 5 mM concentrations in DMSO; after their dilution, no more than 1% DMSO was present in any assay; enzyme activity was monitored for the presence of Tween 20 and DMSO.

### 5.10. Cell Cycle Distribution

The distribution of cells in the major phases of the cell cycle was measured by flow cytometry, which allowed both the identification of DNA stained with the fluorescent dye propidium iodide and accurate analysis of the DNA histogram in the cell population. The cell monolayer at specific time points was washed with PBS and trypsinised. The resulting cell suspension was then centrifuged for 5 min at 1000 rpm, after which the supernatant was removed. The resulting cell pellet was washed twice with PBS. Cells were resuspended in PBS and then mixed with 70% cold ethanol. Immediately before measurement, the cell suspension in ethanol was centrifuged for 5 min at 2000 rpm, then the cell pellet was washed with PBS, and after centrifugation for 5 min at 1000 rpm, resuspended in PBS containing propidium iodide and RNAse A at final concentrations of 75 µM and 20 µg/mL, respectively. This was followed by a 30 min incubation in complete darkness at 37 °C. Stained cells were analyzed with a flow cytometer (Becton-Dickinson, East Rutherford, NJ, USA) and data analysis was performed with the FlowJo cytology software.

### 5.11. Clonogenicity Assay

To determine the effect of tested compounds on the clonogenicity of cancer cells, HepG2 and HUH7 cells were split into single cells and plated at 500 cells/mL in DMEM complete medium onto regular cell culture dishes. Cells were cultured for 72 h in the presence of tested compounds at a concentration of 25 µM, then the growth medium was replaced with another fresh medium and the cells were cultured for another 14 days. Briefly, after washing with PBS, cells were fixed with methanol for 1 h. Cells were then stained with 0.1% crystal violet in 35% ethanol for 20 min at room temperature. The plates were rinsed 5 to 10 times with water until no dye was detected during rinsing. After air drying, colonies were photographed and counted for comparison of the number of colonies as described elsewhere [57].

### 5.12. ROS Measurement

The non-fluorescent form of the dye H_2_DCF-DA (reduced form of fluorescein) penetrates into the cell and after hydrolysis of the acetate groups by intracellular esterases and oxidation by ROS present in the cell, transforms into a highly fluorescent product, DCF (2′,7′-dichlorofluorescein) [58]. To analyze the kinetics of ROS generation by tested compounds, cells were incubated with them for 2 h. After incubation, the cell monolayer was washed with HBSS solution, cells were incubated with a fluorescent probe (at a final concentration of 5 µM), then the kinetics of ROS generation in the culture was monitored for 3 h by measuring the fluorescence of the oxidized probe every 15 min at λ_ex_ = 485 nm/λ_em_ = 538 nm (fluorescence was measured on a Fluoroscan Ascent FL microplate reader, Labsystem, Vantaa, Finland). The results are presented as a percentage; the control value was taken as 100%.

### 5.13. RNS Measurement

For the measurement of intracellular RNS, we used a simple and fast method involving flow cytometry and nitric oxide-specific probe, DAF-FM-DA (4-amino-5-methylamino-2′,7′-difluorofluorescein diacetate). After cell penetration and hydrolysis by intracellular esterases, DAF-FM is released and reacts with nitric oxide to form a highly fluorescent triazolo-fluorescein analog, DAF-FM-T [59]. To analyze the kinetics of RNS production by tested compounds, cells were incubated with them for 2 h. After incubation, the cell monolayer was washed with HBSS solution, cells were incubated with the fluorescent probe (at a final concentration of 5 µM), then the kinetics of RNS production in the culture was monitored for 3 h by measuring the fluorescence of the oxidized probe every 15 min (fluorescence was measured on a Fluoroscan Ascent FL microplate reader at λ_ex_ = 485 nm/λ_em_ = 538 nm). Results are presented in %; the control value was taken as 100%.

### 5.14. Fast Halo Assay

Assessment of DNA damage in hepatocellular cancer cells was performed by fast halo assay (FHA) according to the procedure of Sestili et al. [60]. Briefly, at appropriate time points, control and treated cells were released from monolayers by trypsinization, resuspended in PBS, and washed twice. The cell suspension was added to the low-melting-point agarose solution and a small amount of the suspension was applied to a glass slide precoated with standard agarose and then covered with a coverslip. After the agarose had solidified, the coverslip was removed, and the slides were incubated for 15 min in 0.3 M NaOH at room temperature. During the last 5 min of incubation ethidium bromide was added. After incubation, slides were washed and destained for 5 min in distilled water and immediately examined under a fluorescence microscope (Olympus IX70). Images were analysed using ImageJ software and HaloJ plugin [61]. The results are expressed after calculating the nuclear diffusion factor (NDF), which represents the ratio between the total area of the halo plus the nucleus and that of the nucleus.

### 5.15. Autophagy Assessment

Autophagy induction was determined by measuring monodansylcadaverine (MDC) fluorescence according to a modified method described by Vázquez and Colombo [62]. Cells were seeded into black 96-well microplates. After 24 h, tested compounds were added and cells were incubated for another 24 h, 48 h or 72 h. At the end of incubation, cells were incubated with MDC at a final concentration of 50 µM for 15 min at 37 °C. After this time, the probe was removed and cells were lysed by adding lysis buffer (10.0 mM Tris-HCl, pH 8.0, 0.1% Triton X-100). Intracellular fluorescence intensity of MDC was measured with a Fluoroscan Ascent FL microplate reader at λ_ex_ = 335 nm/λ_em_ = 512 nm. To normalize the number of cells per sample, the amount of DNA was determined by adding ethidium bromide (EB) at a final concentration of 0.2 mM to each well after measuring the MDC fluorescence. EB fluorescence was measured at λ_ex_ = 530 nm/λ_em_ = 590 nm. The results were finally shown as the ratio of MCD/EB fluorescence.

### 5.16. Statistical Analysis

All data are expressed as mean ± SD and presented as a percentage of control (untreated cells) taken as 100%, *n* = 3 (or more). The sample size was estimated for type I and type II statistical errors of 0.05 and 0.8, respectively. The normality of the data was tested with the Shapiro–Wilk test and the homogeneity of variance was verified with the Levene’s or Brown-Forsythe tests. The significance of differences between pairs of means was estimated using one-way ANOVA and Tukey’s post-hoc test. The significance of differences between data received from the clonogenic assay was analyzed with the Chi-square test. Survival curves for **2a**–**2d** compounds and their IC_50_ values were obtained with using Graph-Pad Prism program. The post-hoc power of the used tests was checked for each analysis. A statistical power below 80% was considered an invalid outcome, and constructive conclusions were not formulated. All statistics were calculated using the statistical software package STATISTICA (StatSoft, Tulsa, OK, USA) or GraphPad Prism.

## Figures and Tables

**Table 1:** The IC_50_ values for **2a**–**2d** obtained for HUH7 and HepG2 cell lines as well as the impact of **2a–2d** used at IC_50_ on the colony forming ability (clonogenicity).

Compound	Cytotoxicity IC_50_ (µM)		Clonogenicity (% of Ctrl)	
	HUH7	HepG2	HUH7	HepG2
**2a**	57.1 ± 2.9	15.0 ± 0.8	22.5 ± 3.1	15.8 ± 1.4
**2b**	58.3 ± 2.9	2.8 ± 0.1	25.5 ± 3.6	9.9 ± 0.9
**2c**	213.7 ± 10.7	302.1 ± 15.1	77.5 ± 4.5	83.4 ± 3.5
**2d**	105.3 ± 5.3	16.3 ± 0.3	40.0 ± 4.1	13.0 ± 1.4
**3BP**	No cytotoxic properties up to 500 µM			
**2DG**	No cytotoxic properties up to 500 µM			

Cells were incubated with the tested compounds for 72 h; data are expressed as mean (µM) and SD, *n* = 3; IC_50_ values were received based on Graph-Pad Prism software calculations. Clonogenicity: HUH7 and HepG2 cells were incubated with **2a**–**2d** for 72 h at their IC_50_; data are expressed as mean (% of control cells treated as 100%) and SD, *n* = 3; Chi-square analysis was performed to evaluate the significance of differences between control and treated cells as well as between cell lines and used chemicals; statistical analysis for HUH7: *p* < 0.05: **2a** vs. **2d**, **2b** vs. **2d**; *p* < 0.01: control vs. **2c**, **2c** vs. **2d**; *p* < 0.001: control vs. **2a**, **2b** and **2d**; for HepG2: *p* < 0.05: control vs. **2c**; **2a** vs. **2b**; *p* < 0.001: control vs. **2a**, **2b** and **2d**, **2c** vs. **2a**, **2b** and **2d**; significant differences between used cell lines and tested agents: *p* < 0.05: (**2a**) HUH7 vs. HepG2; *p* < 0.01: (**2b**) HUH7 vs. HepG2; *p* < 0.001 (**2d**) HUH7 vs. HepG2.
